# Hepatitis C Virus Screening and Emergency Department Length of Stay

**DOI:** 10.1371/journal.pone.0164831

**Published:** 2016-10-19

**Authors:** Douglas A. E. White, Erik S. Anderson, Sarah K. Pfeil, Laura J. Deering, Tamara Todorovic, Tarak K. Trivedi

**Affiliations:** 1 Department of Emergency Medicine, Alameda Health System, Highland Hospital, Oakland, CA, United States of America; 2 Department of Emergency Medicine, Stanford University, Palo Alto, CA, United States of America; Yokohama City University, JAPAN

## Abstract

**Background:**

Recent studies demonstrate high rates of previously undiagnosed hepatitis C virus (HCV) infection among patients screened in urban emergency departments (ED). Experts caution, however, that public health interventions, such as screening for infectious diseases, must not interfere with the primary mission of EDs to provide timely acute care. Increases in ED length of stay (LOS) have been associated with decreased quality of ED care.

**Objective:**

In this study, we assess the influence of an integrated HCV screening protocol on ED LOS.

**Methods:**

This was a retrospective cohort study analyzing timestamp data for all discharged patients over a 1-year period. The primary outcome compared the median LOS in minutes between patients who completed HCV screening and those who did not. Further analysis compared LOS for HCV screening by whether or not complete blood count (CBC) testing was conducted.

**Results:**

Of 69,639 visits, 2,864 (4%) had HCV screening tests completed and 272 (9.5%) were antibody positive. The median LOS for visits that included HCV screening was greater than visits that did not include screening (151 versus 119 minutes, *P* < 0.001). Among the subset of visits in which CBC testing was conducted, there was no significant difference in median LOS between visits that also included HCV screening and those that did not (240 versus 242 minutes, *P* = 0.68).

**Conclusion:**

Integrated HCV screening modestly prolongs ED LOS. However, among patients undergoing other blood tests, screening had no effect on LOS. Programs may consider routinely offering HCV screening to patients who are undergoing laboratory testing.

## Introduction

### Background

The Centers for Disease Control and Prevention recommend targeted hepatitis C virus (HCV) screening in health care settings for a “birth cohort” of patients born between 1945–1965, as well as those with identifiable risk factors, such as injection drug use.[1] Emergency departments (EDs) that have implemented HCV screening programs have identified a prevalence of HCV antibody positivity of approximately 10%.[2–6] The surprisingly high rates of undiagnosed HCV infection among ED patients highlights the import role EDs could play in combating the HCV epidemic through screening, disease identification, and linkage to care and treatment.[7] Experts caution, however, that public health interventions such as screening for infectious diseases must not interfere with the primary mission of EDs to provide timely acute care.[8]

In April 2014, we integrated triage nurse HCV screening into ED clinical operations, utilizing a laboratory-based testing protocol and native staffing to offer, perform, and disclose results.[2] Because of concerns that HCV screening would increase ED length of stay (LOS) our protocol did not require patients to wait for the results of their HCV tests prior to discharge.

### Importance

Little is known about how screening for infectious diseases, such as HIV and HCV, impacts LOS. Emergency department LOS is a well-accepted surrogate marker for crowding and has been associated with poor clinical outcomes.[9–11] Although Coeller et al. demonstrated that ED LOS is negligibly affected by HIV screening, the generalizability of their findings is limited because a parallel staffing model and rapid oral swabs were utilized for testing.[12] To our knowledge, no such literature exists for HCV screening and for models that utilize native staffing and standard blood-based laboratory testing procedures.

### Goals of This Investigation

The objective of this study is to assess the impact of a streamlined and integrated HCV screening protocol on ED LOS for patients discharged from the ED.

## Methods

### Study Design

In this retrospective cohort study we assess the LOS for all patients discharged from the ED, and compared timestamp data between patients completing HCV screening and those who did not. This study received approval from the Alameda Health System Institutional Review Board. This study applied for and received a waiver of written informed consent. Patient records were anonymized and de-identified prior to analysis.

### Setting

Alameda Health System—Highland Hospital is an urban teaching hospital and trauma center with an accredited emergency medicine residency program in Oakland, California. The annual ED census is approximately 80,000 patients; 45% are black, 44% female, and 85% have public insurance. Triage takes place in a non-private setting and patients are designated for treatment in either the Main ED (70%) or the Fast Track (FT) (30%) part of the ED. The 2 sites share a common staff consisting of attending and resident physicians, physician assistants, nurses, and technicians. All blood tests are sent by tube system and processed immediately by the laboratory. Anti-HCV antibody tests are performed on the Abbott Architect (Abbott Laboratories, Abbott Park, IL) with a laboratory median turn-around time of 70 minutes (interquartile range [IQR)] 59 to 94).[13] The median laboratory turn-around time for complete blood count (CBC) testing is 22 minutes (IQR 15 to 33).

### Data Collection and Processing

Patient-specific laboratory data, including HCV antibody results and whether a CBC test was performed (a surrogate for any other laboratory test being completed), was captured from the laboratory electronic medical record (EMR) (Novius, Siemens Corporation). Patient demographics and timestamped triage and discharge times were captured from the ED EMR (Wellsoft Corporation, Somerset, NJ). These data were linked via patient account numbers unique to each visit and entered into a spreadsheet (Microsoft Excel 2007; Microsoft Corporation, Redmond, WA). Patient identifying information was then removed and each visit was assigned a unique study number. Any missing data was addressed by individual chart review by study investigators.

### Outcome Measures

The primary outcome was the comparison of median LOS in minutes among discharged patients who completed HCV screening and those who did not. We chose to exclude patients that were admitted to the hospital as HCV testing ought to have no effect on patients boarding in the ED awaiting inpatient beds. We additionally stratified patients based on location of care (Main ED versus FT) and whether or not other laboratory testing was done (CBC versus no CBC). Length of stay was defined as the time between the triage timestamp (recorded when a new triage template was opened) and the discharge timestamp (recorded when discharge instructions were printed).

### Primary Data Analysis

Unless otherwise specified, visit level data are presented and descriptive analyses performed for all variables. Continuous data (including LOS) is reported as median with IQR or proportions as percentages with 95% confidence intervals (CIs). Only patients with complete time interval data were analyzed. Because LOS is positively skewed with significant outliers, medians and IQRs were compared using the Wilcoxon rank sum test. The influence of other laboratory testing and location of care on LOS was explored using bivariate analyses. No *a priori* sample size calculation was performed because this was a retrospective, descriptive analysis conceived after implementation of a clinical protocol. All statistical analyses were performed using Stata version 13 (StataCorp LP, College Station, TX).

## Results

### Characteristics of Study Subjects

**Fig 1:**
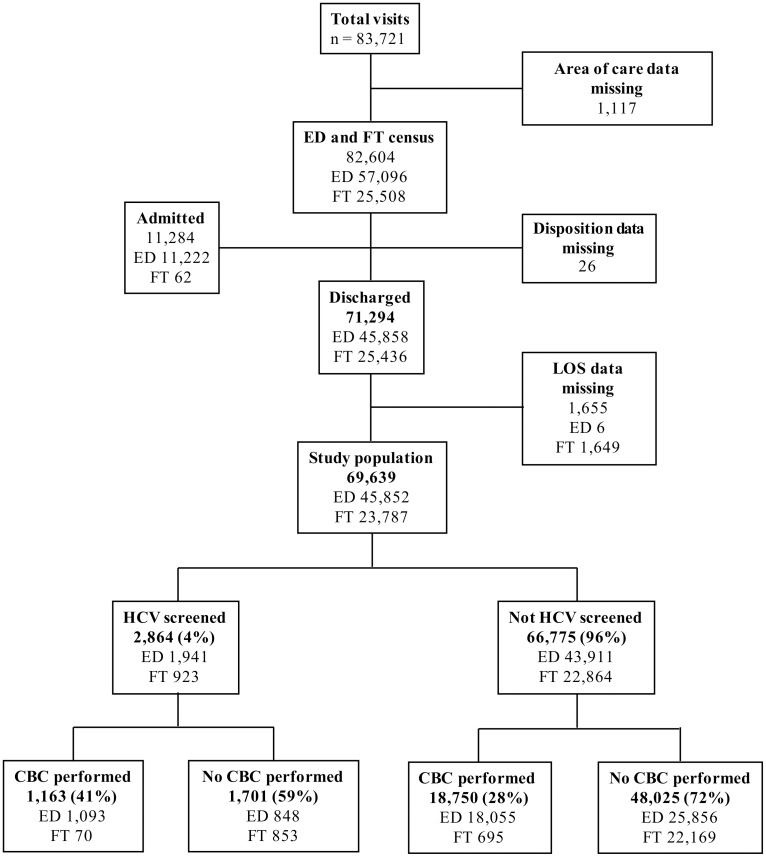
Characteristics of study population. ED = Emergency Department; FT = Fast Track/Urgent Care; HCV = Hepatitis C virus.

From April 2014 through March 2015, there were 83,721 visits to the ED. The final study sample consisted of 69,639 visits with complete LOS data, in which location of care was known and the patient was discharged. (Fig 1) Only discharged patients were included in the analysis.

There were 44,027 unique patients with a mean age of 40.2 (SD 15.0); 48% were female, 39% black, 13% white, 34% Hispanic, and 4% were homeless. Of the 69,639 visits, 45,852 (66%) were triaged to the Main ED and 23,787 (34%) were triaged to the FT; and 19,913 (29%) patients had CBCs performed. Patients seen in the Main ED were more likely to have CBCs performed (42%) than patients in the FT (3%) (*P*<0.001).

There were 2,864 HCV screening tests completed, of which 272 (9.5%) were antibody positive. Hepatitis C virus screening was significantly associated with CBC testing, occurring in 5.8% of patients who also had a CBC completed versus 3.4% of those who did not (odds ratio [OR] 1.75, 95% CI 1.62 to 1.89). Hepatitis C virus screening was also significantly associated with the location of care being the Main ED as compared to the FT (4.2% versus 3.9%, OR 1.09, 95% CI 1.01 to 1.19). There was no difference in the rate of HCV antibody positivity, however, between patients screened in the Main ED (9.9%) versus the FT (8.7%) (*P* = 0.31) or between those who were also CBC tested (10.0%) versus those who were not CBC tested (9.2%) (*P* = 0.47).

### Main Results

#### Overall length of stay

The median LOS among all 69,639 visits to the Main ED and FT was 120 minutes (IQR 48 to 223). Visits that occurred in the Main ED had a significantly longer median LOS (179 minutes, IQR 108 to 278) than those in the FT (39 minutes, IQR 21 to 72) (*P*<0.001); and visits that included CBC testing had a longer median LOS (242 minutes, IQR 170 to 347) than those without CBC testing (77 minutes, IQR 34 to 151) (*P*<0.001).

**Fig 2:**
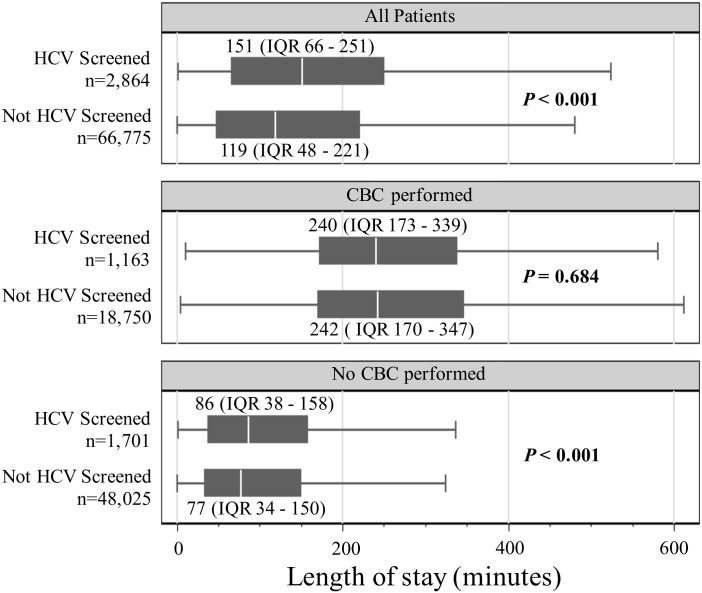
Median length of stay for all patient visits (Main ED and FT). ED = Emergency Department; FT = Fast Track; HCV = hepatitis C virus; IQR = interquartile range; CBC = complete blood count.

The median LOS for all visits to the Main ED and FT that included HCV screening was 151 minutes (IQR 66 to 251) compared with 119 minutes (IQR 48 to 221) for all visits that did not include HCV screening (*P*<0.001). Among all visits in which a CBC was performed, there was no significant difference in median LOS between the visits that also included HCV screening (240 minutes, IQR 173 to 339) and the visits that did not (242 minutes, IQR 170 to 347) (*P* = 0.68). However, among all visits in which CBC testing was not performed, there was a significant difference in median LOS between the visits that included HCV screening (86 minutes, IQR 38 to 158) and the visits that did not (77 minutes, IQR 34 to 150) (*P*<0.001). (Fig 2)

#### Length of stay main emergency department visits

**Fig 3:**
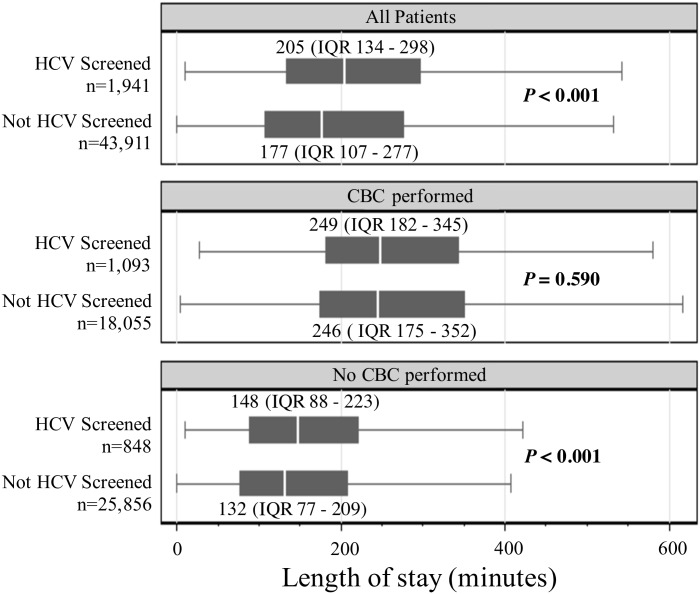
Median length of stay for patient visits in the Main ED. ED = Emergency Department; HCV = Hepatitis C virus; IQR = interquartile range; CBC = complete blood count.

Among visits to the Main ED, there was a significant difference in median LOS between the visits that included HCV screening (205 minutes, IQR 134 to 298) and those that did not (177 minutes, IQR 107 to 277) (*P*<0.001). Among visits in which a CBC was performed there was no significant difference in median LOS between the visits that also included HCV screening (249 minutes, IQR 182 to 345) and the visits that did not (246 minutes, IQR 175 to 352) (*P* = 0.590). However, among visits in which CBC testing was not performed, there was a significant difference in median LOS between the visits that included HCV screening (148 minutes, IQR 88 to 223) and the visits that did not (132 minutes, IQR 77 to 209) (*P*<0.001). (Fig 3)

#### Length of stay fast track visits

**Fig 4:**
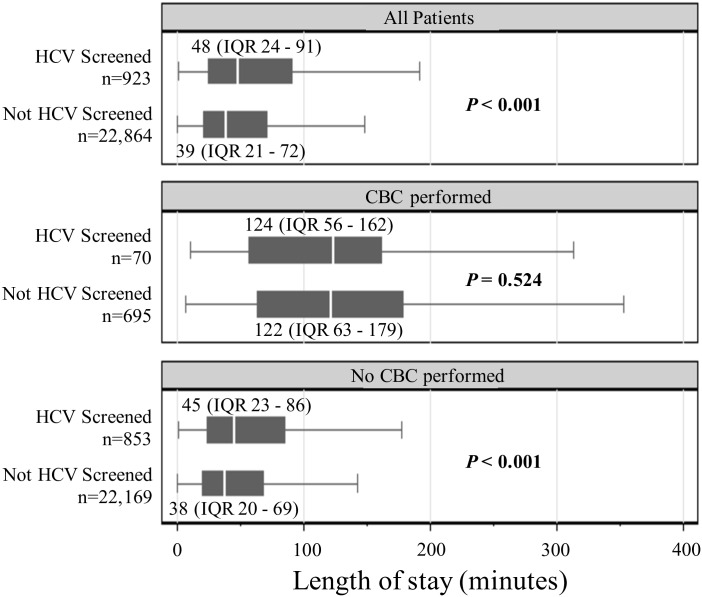
Median length of stay for patient visits in the Fast Track. HCV = hepatitis C virus; IQR = interquartile range; CBC = complete blood count.

Among FT visits, there was smaller but also significant difference in median LOS between the visits that included HCV screening (48 minutes, IQR 24 to 91) and those that did (39 minutes, IQR 21 to 72) (*P*<0.001). Among FT visits in which a CBC was performed, there was no significant difference in median LOS between the visits that also included HCV screening (124 minutes, IQR 56 to 162) and the visits that did not (122 minutes, IQR 63 to 179) (*P* = 0.524). However, among FT visits in which CBC testing was not performed, there was a significant difference in median LOS between the visits that included HCV screening (45 minutes, IQR 23 to 86) and the visits that did not (38 minutes, IQR 20 to 69) (*P*<0.001). (Fig 4)

## Limitations

This study was carried out in a single center, urban ED with a site-specific protocol that may limit the generalizability of our findings. Importantly, we did not require patients to wait for HCV test results prior to discharge. The influence of HCV screening on LOS likely would have been greater if we had mandated that patients wait for test results. We did not evaluate, nor control for, other factors that may influence LOS, such as acuity, time of day, other diagnostic interventions, and ED staffing patterns. Lastly, using CBC testing as the sole surrogate for other blood tests being performed misses some such visits.

## Discussion

Emergency department screening for diseases such as HIV and HCV is not without controversy. Critics argue that EDs already struggle to provide timely acute care, and that undertaking further preventative care or public health interventions may interfere with this primary mission.[8] The benefits of screening must therefore be weighed against any negative impact on metrics that may interfere with this mission, such as LOS.

In this study we show that in an urban, academic ED, HCV screening has a modest effect on LOS. Prior work has shown that ED-based HIV screening utilizing a parallel staffing model resulted in visits that were 2% longer than when HIV screening was not completed.[12] This is the first study to examine the influence of HCV screening on ED LOS using existing staff and laboratory resources. We demonstrate that the increased LOS seen with HCV screening is limited to the screened patients who did not otherwise have blood tests performed. It is primarily in these visits when blood testing would not otherwise be performed, which includes over 95% of patients seen in our FT but just over half of Main ED visits, that the impact of an increase in LOS must be weighed against the public health benefit of screening.

We believe the public health benefit of HCV screening outweighs the modest influence on LOS. Hepatitis C virus infection affects more than 3 million U.S. persons, is the leading cause of hepatocellular carcinoma, end-stage liver disease, and liver transplantation.[1] It is estimated that nearly half of patients with HCV infection do not know their diagnosis.[14] Emergency departments are a potentially important venue for HCV screening, as studies demonstrate high rates of previously undiagnosed infection among screened ED patients.[2–6] Importantly, advances in antiviral treatments have made what was recently thought of as an untreatable disease, curable.

Our data demonstrates that when HCV screening occurs during visits when other blood tests are performed, the addition of the screening test has no influence on LOS. Unfortunately, of the nearly 20,000 visits during our study period in which CBC testing was performed, concomitant HCV screening occurred less than 6% of the time. The failure to screen patients who had blood drawn for other tests represents a missed screening opportunity. If we applied a protocol of screening all patients in the birth cohort who underwent CBC testing in our study population, we estimate that approximately 1,100 patients would be diagnosed HCV antibody positive. This yield of HCV antibody positive would be almost four-fold higher than our existing protocol, and would likely have no influence on ED LOS. While some patients may decline screening, Geren et al. report that a similar protocol for ED HIV screening resulted in acceptance rates of over 90% in patients already undergoing venipuncture.[15] Such an integrated protocol may also alleviate ED administrative concerns about a screening program’s adverse impact on LOS, and serve as a generalizable model for ED-based HCV screening dissemination.

In conclusion, we show that an integrated HCV screening program modestly prolongs overall LOS throughout the ED. However, among patients undergoing other blood tests, HCV screening had no significant effect on LOS. Emergency departments must consider whether the public health benefit of screening justifies the impact on quality metrics, such as LOS, which has been shown to influence the ability to provide timely acute care. Future programs should consider routinely offering HCV screening to patients who are undergoing laboratory testing.

## References

[1] Smith BD, Morgan RL, Beckett GA, Falck-Ytter Y. Recommendations for the identification of chronic hepatitis C virus infection among persons born during 1945–1965. *MMWR Recomm Rep*. 2012;61:1–36. 22895429

[2] White DAE, Anderson ES, Pfeil SK, Trivedi TK, Alter HJ. Results of a rapid hepatitis C virus screening and diagnostic testing program in an urban emergency department. *Ann Emerg Med*. 1 2016;67(1):119–128. 10.1016/j.annemergmed.2015.06.023 26253712

[3] Galbraith JW, Franco RA, Donnelly JP, Rodgers J, Morgan J, Viles A, et al Unrecognized chronic hepatitis C virus infection among baby boomers in the emergency department. *Hepatology*. 9 2014:n/a–n/a. 10.1002/hep.27410 25179527

[4] Merchant RC, Baird JR, Liu T, Taylor LE. HCV among The Miriam Hospital and Rhode Island Hospital Adult ED Patients. *R I Med J*. 2014;97(7):35–39. PMC4349365 24983020

[5] Allison WE, Chiang W, Rubin A, O'Donnel L, Saldivar MA, Maurantonio M, et al Hepatitis C virus infection in the 1945–1965 birth cohort (baby boomers) in a large urban Emergency Department. *Amer J Emerg Med*. 2015. In Press. 10.1016/j.ajem.2015.12.072 26809931

[6] Anderson ES, Pfeil SK, Deering L, Todorovic T, Lippert S, White DAE. High-impact hepatitis C virus testing for injection drug users in an urban ED. *Am J Emerg Med*. 2016 In Press. 10.1016/j.ajem.2016.03.004 27037135

[7] Galbraith JW. Hepatitis C virus sreening: an important public health opportunity for United States emergency departments. *Ann Emerg Med*. 2016;67(1):129–130. 10.1016/j.annemergmed.2015.08.002 26342899

[8] Moran GJ, Talan DA. Processes and models for HIV screening in the emergency department: can and should we do this? *Ann Emerg Med*. 2011;58(S):S172–S173. 10.1016/j.annemergmed.2011.03.044 21684400

[9] Herring A, Wilper A, Himmelstein DU, Woolhandler S, Espinola JA, Brown DF, et al Increasing length of stay among adult visits to U.S. emergency departments, 2001–2005. *Acad Emerg Med*. 2009;16(7):609–616. 10.1111/j.1553-2712.2009.00428.x 19538503

[10] Kocher KE, Meurer WJ, Desmond JS, Nallamothu BK. Effect of testing and treatment on emergency department length of stay using a national database. *Acad Emerg Med*. 2012;19(5):525–534. 10.1111/j.1553-2712.2012.01353.x 22594356

[11] Hollander JE, Pines JM. The Emergency Department Paradox: The longer you stay, the less care you get. *Ann Emerg Med*. 2007;50(5)497–499. 10.1016/j.annemergmed.2007.05.002 17583380

[12] Coeller N, Kuo I, Brown J. Nontargeted rapid human immunodeficiency virus screening provided by dedicated personnel does not adversely affect emergency department length of stay. *Acad Emerg Med*. 2011;18(7):708–713. 10.1111/j.1553-2712.2011.01100.x 21729185

[13] White DAE, Anderson ES, Pfeil SK, Trivedi TK. Hepatitis C Virus Antibody Testing: Result Availability at Time of Discharge for Emergency Department Patients. *J Acquir Immune Defic Syndr*. 2016;71(3):e82–84. 10.1097/QAI.0000000000000887 26536320 PMC4770363

[14] Denniston MM, Klevens RM, McQuillan GM, Jiles RB. Awareness of infection, knowledge of hepatitis C, and medical follow-up among individuals testing positive for hepatitis C: National Health and Nutrition Examination Survey 2001–2008. *Hepatology*. 2012 6;55(6):1652–61. 10.1002/hep.25556 22213025 PMC4586034

[15] Geren KI, Lovecchio F, Knight J, Fromm R, Moore E, Tomlinson C, et al Identification of acute HIV infection using fourth-generation testing in an opt-out emergency department screening program. *Ann Emerg Med*. 2014;64(5):537–546. 10.1016/j.annemergmed.2014.05.021. 24970245

